# Editorial: Plant stress – a threat to food security

**DOI:** 10.3389/fpls.2025.1631524

**Published:** 2025-06-23

**Authors:** Jagna Chmielowska-Bąk, Yuriy E. Kolupaev, Yaroslav B. Blume

**Affiliations:** ^1^ Department of Plant Ecophysiology, Faculty of Biology, Adam Mickiewicz University, Poznań, Poland; ^2^ Yuriev Plant Production Institute, National Academy of Agrarian Sciences of Ukraine, Kharkiv, Ukraine; ^3^ Institute of Food Biotechnology and Genomics, National Academy of Sciences of Ukraine, Kyiv, Ukraine

**Keywords:** abiotic stress, drought, salt stress, heavy metals, cell signaling, gene expression, phytohormones, nanomaterials

The intensification of abiotic stress impacts on plants is becoming one of the main threats to global food security. According to predictions estimates, in the nearby future, the negative effects of abiotic stress factors on crop production may lead to food shortage for 1.8 billion people (Nyaupane et al., 2024). Drought, high temperatures, and soil salinization are the primary factors limiting yield growth and posing food security risks. Currently, drought-attributed crop losses range from 30% to 90% (Dietz et al., 2021), exceeding the total losses caused by all pathogens combined (Gupta et al., 2020). Another factor, often affecting plants in the same regions as drought, is soil salinization, which affects at least 20% of all irrigated areas (Mushtaq et al., 2021). Alongside drought and salinization, the impact of extremely high temperatures on food crops is also intensifying. It is predicted that the mean global temperature may be 1.8–4.0°C higher by the end of this century than in 2000 (Munaweera et al., 2022). These climate changes are, at least in part, associated with anthropogenic factors. The impact of heavy metals, metalloids, and other xenobiotics on plants and other groups of organisms is even more clearly related to human activities.

At the same time, plants possess enormous adaptive potentials, which are being continuously elucidated due to advances in studying the mechanisms of stress signal perception and transduction to the genetic apparatus, transcriptomic and proteomic changes, post-translational protein modifications, and epigenetic regulatory mechanisms. The Research Topic entitled *“*Plant Stress – A Threat to Food Security*”* brings together studies focused both on uncovering new fundamental mechanisms of adaptation, including functioning of macromolecules and signal-regulatory compounds, and on expanding the practical use of new bioregulators.

Several articles in this Research Topic are devoted to mechanisms of plant adaptation to drought, salinity, high temperatures, and induction of resistance to these factors through exogenous hormones and micronutrient nanoparticles. For example, Zhang et al. studied the heat shock protein 20 (Hsp20) gene family in *Lactuca sativa*. One of the most interesting findings was the identification of three types of cis-elements within the LsHsp20 family: (1) stress-responsive, (2) hormone-responsive, and (3) development-related. This expands our understanding of the Hsp20 functions in plants.

New hormones and compounds with hormone-like activities (Feng et al., 2024) hold significant potentials for enhancing plant tolerance to drought and associated stressors. Positive effects of one form of auxin (indole-3-butyric acid) were demonstrated by Zhou et al. in experiments on rice plants subjected to salt stress. The authors attribute the observed increase in shoot and root growth to modulated carbohydrate metabolism and activated syntheses of several secondary metabolites with stress-protective capacities. In the study by Cavalcante et al., the effectiveness of salicylic acid as a drought tolerance inducer in an important forage crop, *Vigna unguiculata*, was examined. In field experiments, the authors showed that salicylic acid enhanced antioxidant activity and osmolyte synthesis under drought, increasing the performance, although these effects appeared to be cultivar-specific. The need to pay close attention to species- and cultivar-specific peculiarities of adaptation of domestic plants to drought is also emphasized in the article by Zhou et al. The authors conducted the first bibliometric analysis of publications on drought tolerance of *Medicago sativa*. They concluded that for many years, two main directions have been prioritized: research into the mechanisms of alfalfa’s response to drought and development of technological approaches to boost drought tolerance.

The review by Bao et al. analyses the potential use of nanomaterials to enhance plant tolerance to drought and high temperatures, with a focus on the activation of antioxidant systems by nanoparticles. Although antioxidant effects have been observed in plants treated with nanomaterials containing Ag, Zn, Ti, Se, and Mn compounds, the authors emphasize that the potential accumulation of these nanomaterials in the edible parts of agricultural plants may pose a threat to human health. Therefore, practical applications of nanomaterials must be preceded by thorough toxicological and ecological studies (Ekner-Grzyb et al., 2022). Nevertheless, it was revealed that treatment of plants with nanomaterials could considerably mitigate effects of toxic elements on plants. A striking example of the promising potential of such research is given in the study by Zeeshan et al., where zinc oxide nanoparticles (ZnO-NPs) significantly improved the growth of soybean plants exposed to arsenic. Co-treatment with ZnO-NPs led to hampered As uptake accompanied by enhanced growth and photosynthesis parameters. These effects could be a result of ZnO-NPs-dependent modulation of genes expression. The transcriptomic data revealed distinct expression of genes engaged in As transport, stress response, signaling and phytohormone metabolism under ZnO-NPs supplementation.

The topic of plant adaptation to toxicants is further developed in the review by El-Sappah et al. In particular, the review focuses on transporter proteins that transport heavy metal ions into vacuoles and on regulation of expression of genes encoding these proteins. This article describes the sources of metal contamination and pathways leading to their uptake by plants, transport within cells and distinct organs and activated defense mechanisms leading to their sequestration. In addition to the negative effects of metals/metalloids, some beneficial roles are presented, e.g. cofactors, metabolism modulators and anti-herbivore agents. Nonetheless, metals/metalloids in excess are harmful to plants and, thus, methods for alleviation of their toxicity are intensively studied.

Two other review articles are devoted to the general mechanisms of plant adaptation. In their short review, Wang et al. summarize recent findings on the role of histone acetylation in the regulation of plant responses to different abiotic stressors. The authors discuss technical aspects of studying this process and highlight the promising potential of evaluating histone acetylation’s role in complex responses to multiple simultaneous stressors where histone acetyltransferases interact with several transcription factors and protein complexes, which are often co-regulated by other modifications such as methylation.

Close attention has been recently paid to the role in plant adaptation of several compounds that combine some properties of stress-related metabolites and phytohormones and/or components of signaling pathways. These include, in particular, gaseous signaling molecules (gasotransmitters) (Kolupaev et al., 2022) and melatonin, a hormone, which is well-studied in human and animal physiology but also synthesized in significant amounts by plants (Aghdam and Arnao, 2024). The review by Kolupaev et al. summarizes data indicating the involvement of nitric oxide, hydrogen sulfide, and carbon monoxide in melatonin’s stress-protective functions in plants. It also discusses how melatonin modulates one of the most important effects of gasotransmitters and reactive oxygen species—post-translational protein modifications.

In conclusion, it should be noted that, despite its high relevance, the presented selection of articles cannot fully reflect the complexity of solving plant adaptation challenges in the context of food security. Nonetheless, the effectiveness of scientific efforts can be increased through interdisciplinary work, bridging studies at the molecular and cellular levels with studies on whole plants followed by translation of discovered effects into new biotechnologies.
